# Associations between Th17-related inflammatory cytokines and asthma in adults: A Case-Control Study

**DOI:** 10.1038/s41598-017-15570-8

**Published:** 2017-11-14

**Authors:** Ting Zhou, Xiji Huang, Yun Zhou, Jixuan Ma, Min Zhou, Yuewei Liu, Lili Xiao, Jing Yuan, Jungang Xie, Weihong Chen

**Affiliations:** 1 Department of Occupational and Environmental Health, School of Public Health, Medical College, Wuhan University of Science and Technology, Wuhan, Hubei 430065 China; 2 Hubei Province Key Laboratory of Occupational Hazard Identification and Control, Wuhan University of Science and Technology, Wuhan, Hubei 430065 China; 3 Hubei Center for Disease Control and Prevention, Wuhan, Hubei 430079 China; 4 Department of Occupational & Environmental Health, School of Public Health, Tongji Medical College, Huazhong University of Science and Technology, Wuhan, Hubei 430030 China; 5 Key Laboratory of Environment and Health, Ministry of Education & Ministry of Environmental Protection, and State Key Laboratory of Environmental Health (Incubating), School of Public Health, Tongji Medical College, Huazhong University of Science and Technology, Wuhan, Hubei 430030 China; 6 Department of Respiratory and Critical Care Medicine, Tongji Hospital, Tongji Medical College, Huazhong University of Science and Technology, Wuhan, Hubei 430030 China

## Abstract

Chronic airway inflammation is recognized as an essential process in the pathogenesis of asthma. Cytokine profiles derived from immune and inflammation cells such as T-helper (Th) cells, eosinophilia and neutrophilia are not limited to the Th2 type in asthma. However, little is understood about associations between Th2-low inflammatory cytokine profiles and risk of asthma in adults. A case-control study of 910 adult asthma and 881 healthy controls was conducted. Inflammatory cytokines screening was undertaken by high-throughput protein microarray technology, and Th17-related inflammatory cytokines (IL17A, IL-9, adipsin and CCL11) were finally selected. Associations between these four cytokines and adult asthma risk were analyzed by multivariate logistic regression models. We observed that plasma IL-17A and IL-9 levels were significantly increased in asthmatics when compared with controls. However, the plasma expressions of adipsin and CCL11 in asthmatics were significantly lower than that in health controls. The adjusted ORs (95%CI) of association between IL-17A, IL-9, adipsin and CCL11 expressions and adult asthma were 3.08 (1.91, 4.97), 1.93 (1.41, 2.64), 10.02 (6.99, 14.37) and 3.29 (2.36, 4.59), respectively (all *P*_trend_ < 0.0001). Our results suggested that elevated IL-17A and IL-9 expressions and decreased levels of adipsin and CCL11 were positively associated with adult asthma.

## Introduction

Asthma is one of the most common chronic respiratory diseases, characterized by recurrent and reversible bronchial obstruction, airway hyperresponsiveness and evidence of airway inflammation^1^. It was estimated that approximately 300 million individuals suffered from asthma worldwide, with remarkably increasing incidence in many developing countries^2^. Although the prevalence of asthma in adults (5–10%) is lower than that in children (15–20%), adult asthma is more prone to develop into persistent asthma. Adult asthma is usually accompanied by a lifetime, with an increased rate of decline in lung function^3–5^. Therefore, adult asthma is of particular interest and concern.

Although the exact mechanism of adult asthma is still unclear, chronic airway inflammation such as T-helper (Th) immune, eosinophilic and neutrophilic responses are recognized as cardinal elements in the development of asthma^2,6–8^. Cytokines released by complex interactions of these immune and inflammation cells are thought to be crucial mediators in expanded inflammation and airway obstruction. Many Th2-cell-derived cytokines such as IL-4, IL-5 and IL-13 had the potential to stimulate epithelial cells, smooth muscle cells and fibroblasts that would contribute to airway hyper-responsiveness and remodeling^9–11^. Recently, accumulating evidences have shown that non-Th2 cytokines including IL-8, IL-10 and IL-17 are related with severity of asthma^7,12^. However, little is understood about associations between Th2-low cytokine profiles and risk of asthma in adults.

In this study, we applied high-throughput protein microarray technology to screen 280 plasma cytokines in adult asthmatics and healthy controls, and selected some specific inflammatory cytokines involved in Th2-low inflammation such as Th17-related signaling pathway. Information of occupational exposure, family history of asthma and lifestyle habits including smoking, drinking, and physical activity was collected for each participant. The aim of this study is to investigate associations of Th17-related inflammatory cytokines with asthma risk in adults.

## Results

### Basic characteristics

The basic characteristics of all participants are summarized in Table 1. The age of 1791 participants ranged from 18 to 86 years. The mean age was 45.64 ± 14.11 years for asthmatics and 46.63 ± 14.40 years for controls. Compared with healthy controls, asthmatics were more likely to be less physical activity, drinking, smoking, passive smoking and had a higher percentage of asthma family history and poor sleeping quality (*P* < 0.0001). Lung function parameters such as Forced vital capacity (FVC), FVC%, forced expiratory volume in one second (FEV_1_), FEV_1_%, FEV_1_/FVC and peak expiratory flow (PEF) in asthmatic patients were significantly lower than those in healthy controls (*P* < 0.0001). There were no statistical differences in body mass index (BMI), occupational hazards exposure, keeping pets and planting flowers between asthmatics and healthy controls.

**Table 1:** Basic characteristics of the study population.

Variables	Controls (n = 881)	Asthmatics (n = 910)	*P* value
Age (years)	46.63 ± 14.40	45.64 ± 14.11	0.1424
Female (yes, %)	522 (59.25)	543 (59.67)	0.8565
Education (N, %)			<0.0001
Low (≤9 years)	634 (71.96)	514 (56.48)	
Middle (9~12 years)	178 (20.20)	238 (26.15)	
High (>12 years)	69 (7.83)	158 (17.36)	
Occupational hazards exposure (yes,%)	306 (34.73)	337 (37.03)	0.3105
Physical activity (yes,%)	417 (47.33)	252 (27.69)	<0.0001
Keeping pets (yes,%)	132 (14.98)	153 (16.81)	0.2898
Planting flowers (yes,%)	178 (20.20)	192 (21.10)	0.6401
Family history of asthma (yes,%)	5 (0.57)	100 (10.99)	<0.0001
Smoking status (N,%)			<0.0001
no smoking	647 (73.44)	718 (78.90)	
Former smoking	22 (2.50)	74 (8.13)	
Current smoking	212 (24.06)	118 (12.97)	
Passive smoking (yes,%)	302 (34.28)	217 (23.85)	<0.0001
Drinking (N,%)			<0.0001
No drinking	671 (76.16)	767 (84.29)	
Former drinking	18 (2.04)	49 (5.38)	
Current drinking	192 (21.79)	94 (10.33)	
Sleeping quality (N,%)			<0.0001
Good	538 (61.07)	250 (27.47)	
General	278 (31.56)	442 (48.57)	
Bad	65 (7.38)	218 (23.96)	
BMI (kg/m^2^)	23.22 ± 3.36	23.03 ± 3.48	0.2261
Lung function			
FVC (L)	3.11 ± 0.88	2.91 ± 1.03	<0.0001
FVC (%, predictive value)	102.80 ± 23.75	88.65 ± 23.12	<0.0001
FEV_1_ (L)	2.61 ± 0.69	2.11 ± 0.84	<0.0001
FEV_1_ (%, predictive value)	98.05 ± 24.68	77.82 ± 26.19	<0.0001
FEV_1_/FVC (%)	84.73 ± 8.18	72.52 ± 15.18	<0.0001
PEF (L/s)	5.52 ± 2.13	4.63 ± 2.08	<0.0001

Among asthma patients, symptoms of cough and dyspnea in persistent asthmatics were significantly more severe than those in intermittent asthmatics (*P* < 0.01). However, there is no significant difference in the rate of cyanosis and wheezing sound between intermittent and persistent asthma cases (Table 2).

**Table 2:** Characteristics of clinical symptoms in patients with asthma.

Clinical symptoms and signs	Intermittent asthmatics (n = 537)	Persistent asthmatics (n = 373)	*P* value
Cough status (N, %)			0.0049
No	115 (21.42)	68 (18.23)	
Occasional	204 (37.99)	113 (30.29)	
Chronic	218 (40.60)	192 (51.47)	
Sputum status (N, %)			0.2440
No	178 (33.15)	114 (30.56)	
Occasional	199 (37.06)	128 (34.32)	
Chronic	122 (22.72)	107 (28.69)	
Purulent	38 (7.08)	24 (6.43)	
Dyspnea (N, %)			0.0008
No	179 (33.33)	95 (25.47)	
Mild	175 (32.59)	100 (26.81)	
Moderate	129 (24.02)	115 (30.83)	
Severe	54 (10.06)	63 (16.89)	
Cyanosis (yes,%)	33 (6.15)	24 (6.43)	0.8899
Wheezing sound (yes,%)	149 (27.75)	108 (28.95)	0.6906

### Inflammatory cytokines levels in plasma samples

Table 3 displays the plasma levels of four inflammatory cytokines in the study population. Without adjustment for confounders, plasma IL-17A and IL-9 levels were higher in both persistent and intermittent asthmatics, however, plasma adipsin and CCL11 expressions were significantly lower than those in healthy controls (all *P* < 0.0001). Furthermore, elevated IL-9 levels and decreased expressions of adipsin and CCL11 in persistent asthmatics were statistical significance in comparison with intermittent asthmatics (*P* < 0.05).

**Table 3:** The plasma levels of four kinds of cytokines in the study population.

Cytokines	Controls (n = 881)	Intermittent asthmatics (n = 537)	Persistent asthmatics (n = 373)
IL-17A (pg/ml)	2.27 (0.81, 5.30)	3.91 (2.04, 8.00)*	4.32 (2.75, 7.50)*
IL-9 (pg/ml)	22.69 (7.14, 40.51)	30.26 (12.10, 51.36)*	32.10 (19.05, 64.84)*^#^
Adipsin (ng/ml)	4550.48 (3258.40, 6328.91)	3100.26 (1957.91, 4721.79)*	2154.81 (1570.65, 3675.80)*^#^
CCL11 (pg/ml)	51.39 (36.60, 75.45)	38.07 (27.38, 63.35)*	31.37 (26.59, 51.84)*^#^

Values are median (25^th^~75^th^ quartiles).

*P < 0.0001, compared with control; ^#^ *P* < 0.05,compared with intermittent asthma.

In asthma patients, plasma IL-17A level was negative correlated with adipsin expression (r_s_ = −0.13, *P* < 0.001). Moreover, there were significant correlations between plasma adipsin expression and levels of IL-9 and CCL11 with r_s_ for −0.22 and 0.35, respectively. (all *P* < 0.001) (Table 4). We also found similar relationships between these inflammatory cytokines in intermittent asthma and persistent asthma patients (see Supplementary Tables S1 and S2).

**Table 4:** Correlation coefficients of four cytokines in asthmatics (*P < 0.001).

Cytokines	IL-17A	IL-9	Adipsin	CCL11
IL-17A	1.00			
IL-9	0.06	1.00		
Adipsin	−0.13*	−0.22*	1.00	
CCL11	−0.02	−0.12*	0.35*	1.00

### Association between four inflammatory cytokines and asthmatics

Table 5 presents the adjusted odds ratios (ORs) and 95% confidence (CIs) for asthmatic patients by quartiles of four inflammatory cytokines. After adjustment for age, sex, BMI, education, smoking, passive smoking, drinking, physical activity, family history of asthma, keeping pets, planting flowers and sleeping quality, we observed that ascending IL-17A and IL-9 levels and descending adipsin and CCL11 expressions were significantly related to increased risk of asthma (all *P*_trend_ < 0.0001). The adjusted ORs (95%CI) of IL-17A, IL-9, adipsin and CCL11 for asthmatics in the highest quartile were 3.08 (1.91, 4.97), 1.93 (1.41, 2.64), 10.02 (6.99, 14.37) and 3.29 (2.36, 4.59) in comparison with those in the lowest quartile, respectively. Similar associations were observed between four inflammatory cytokines and persistent asthmatics (all *P*_trend_ < 0.0001). The results show that the adjusted ORs (95%CI) in the highest quartile were 5.79 (2.97, 11.29), 2.56 (1.67, 3.93), 14.61 (8.79, 24.29) and 4.67 (2.96, 7.34) for IL-17A, IL-9, adipsin and CCL11, respectively, when compared with those in the lowest quartile (see Supplementary Table S3).

**Table 5:** Adjusted odds ratios of adult asthma by quartiles of cytokines.

Cytokines	Asthmatics	Controls	Adjusted OR (95% CI)	*P*_trend_
IL-17A (pg/ml)				<0.0001
Q1 (<1.93)	178	270	1.00	
Q2 (1.93~3.75)	196	252	3.55 (2.20, 5.73)	
Q3 (3.75~7.45)	251	196	4.61 (2.77, 7.68)	
Q4 (>7.45)	285	163	3.08 (1.91, 4.97)	
IL-9 (pg/ml)				<0.0001
Q1 (<9.92)	188	259	1.00	
Q2 (9.92~28.69)	171	277	0.84 (0.61, 1.15)	
Q3 (28.69~50.09)	283	165	2.47 (1.80, 3.38)	
Q4 (>50.09)	268	180	1.93 (1.41, 2.64)	
1/Adipsin*10^−5^				<0.0001
Q1 (<18.01)	127	321	1.00	
Q2 (18.01~27.32)	180	268	1.68 (1.22, 2.33)	
Q3 (27.32~44.38)	239	209	2.78 (2.01, 3.84)	
Q4 (>44.38)	364	83	10.02 (6.99, 14.37)	
1/CCL11*10^−3^				<0.0001
Q1 (<14.67)	177	269	1.00	
Q2 (14.67~22.99)	176	274	0.84 (0.61, 1.16)	
Q3 (22.99~34.42)	243	204	1.42 (1.03, 1.95)	
Q4 (>34.42)	314	134	3.29 (2.36, 4.59)	

Adjusted for age, sex, BMI, education, smoking status, passive smoking status, drinking status, physical activity, family history of asthma, keeping pets, planting flowers, sleeping quality.

In addition, the abnormal IL-17A, adipsin and CCL11 levels were significantly associated with adult asthma. The adjusted ORs (95%CI) of abnormal expressions of elevated IL-17A, decreased adipsin and CCL11 for adult asthma patients were 12.50 (5.52, 28.34), 19.55 (11.74, 32.55), and 4.13 (1.78, 9.55) in comparison with normal level, respectively. And there were significant relationships between abnormal levels of elevated IL-17A or decreased adipsin and risk of persistent asthma in adults, with the adjusted ORs (95%CI) of 24.59 (9.69, 62.43) or 37.06 (19.40, 70.78) when compared with normal level, respectively.

## Discussion

Asthma is a chronic airway inflammatory disease characterized by T-helper cell immune response, eosinophilic and/or neutrophilic inflammatory response. Recent studies reported that Th17 cells, known as one of Th2-low cells, have a powerful influence on the pathogenesis of asthma^13–16^. Th17 cytokines such as IL-17A could result in airway remodeling by increasing neutrophil infiltration, mucous cell metaplasia, and smooth muscle mass^13,17,18^. Moreover, higher IL-17A expression was associated with expressions of IL-9, adipsin, and CCL11^19–21^.

Our results found that plasma IL-17A was only significantly negative correlated with adipsin expression, but there were significant correlations between plasma adipsin expression and levels of IL-9 and CCL11. Actually, the process of cytokines interaction is a complex regulatory network in the development of diseases such as asthma. IL-9 is effective in inducing IL-17-producing cells when synergizing with TGF-β. Nevertheless, the IL-9 level could be negatively regulated by IL-23, which is independent of IL-17 signaling^19^. Thus, although IL-17A was not significantly correlated with IL-9 or CCL11, the influence of IL-17A on IL-9 or CCL11 expression may be mediated by adipsin or other regulators.

In this study, we observed that the plasma levels of IL-17A and IL-9 were significantly increased in asthma cases when compared with controls. However, the expressions of adipsin and CCL11 in asthmatics were significantly lower than that in health controls. The relationships became stronger when compared persistent asthmatics with healthy controls.

We found significantly positive association between plasma IL-9 production and adult asthma risk. This is in line with previous studies showing increased IL-9 and IL-9R expressions in lung tissue of asthmatics rather than health controls^22^. Anti-IL-9 monoclonal antibody was effective in treatment of mild asthma^23^. Recently, Elyaman’ study suggested that IL-9 exerts a promoting effect on the differentiation of Th17 cells^19^. It is possible that IL-9 may play a crucial role in development of asthma, combining with Th17-related cytokines.

To our knowledge, this is the first report that plasma adipsin expression was associated with adult asthma. Our results indicated that plasma adipsin levels were statistically lower in asthmatics than healthy controls. Significant negative dose-response relationship was observed between plasma adipsin level and risk of adult asthma. Few previous publications reported adipsin expression in asthmatic patients. Leivo-Korpela and colleagues did not observe difference in adipsin level between female asthmatics and health controls in adults^24^. Fewer sample examples (35 non-smoking female asthmatics) prevented further analysis in their study. The exact mechanism of adipsin in the development of adult asthma is not clear. Adipsin, primarily expressed in monocyte-macrophages and adipocytes, could be obviously inhibited by IL-17^25^. Elevated plasma IL-17A expression in asthma patients may partly contribute to low adipsin level in this study. Furthermore, adipsin, also called as complement factor D, could promote C5 to liberate C5a through alternative complement pathway. C5a overexpression has a critical impact on the pathogenesis of asthma after allergen provocation^26^. Maybe decreased plasma adipsin combined with depressed C5a expression could result in increased risk of asthma. The exact mechanism of adipsin interacting with Th17-related inflammation or complement factors needs to be further confirmed in pathogenesis of adult asthma.

In addition, we observed that decreased plasma CCL11 level was associated with increased risk of adult asthma. Our result is partly supported by Dent’s study, which suggested no difference in sputum CCL11 level between mild asthma and healthy controls^27^. Conversely, some clinical studies indicated that plasma CCL11 expression was higher in asthma attack, uncontrolled and severe asthmatic patients than that in health controls^28–31^. CCL11 (also known as extaxin-1) plays a pivotal role in recruitment of eosinophils into airways in asthma. Adult asthma predominated by eosinophilic response often develops into severe asthma, however, it seems to be mild or moderate asthma when regulated by Th2-low inflammation such as neutrophilic response through Th17 cells pathways^6^. Thus, decreased plasma CCL11 level might be regulated by IL-17 overexpression in mild to moderate adult asthma. Supporting by Schnyder-Candrian’s study, CCL11 expression was largely inhibited by IL-17 administration and could be partly reversed by neutralizing IL-17 antibody treatment in lung tissues of allergic asthma^21^.

This study has several strengths. Firstly, we evaluated associations of Th17-related cytokines with risk of adult asthma in a relatively large sample size. Secondly, total of 280 cytokines expression profiles was measured to select valuable cytokines. Thirdly, potential confounding factors which are supposed to have impacts on asthma were considered in multivariate logistic regression models. However, in this study, there are two limitations. On the one hand, we did not analyze the influence of atopy on the associations between cytokine levels and risk of asthma because of only 9.12% asthma patients caused by atopy in this study. On the other hand, approximately 12.3% asthmatic patients might take medication to control symptoms before they went to hospitals, because they were previously diagnosed as asthma. Accordingly, prospective studies are essential to explore whether atopy or drug medications have influences on the associations of four inflammatory cytokines with risk of asthma in adults.

In conclusion, we found that elevated plasma IL-17A and IL-9 levels and decreased plasma expressions of adipsin and CCL11 were positively associated with asthma risk in adults.

## Methods

### Study population

All subjects were aged more than 18 and living at least 5 years in Wuhan city in China. The asthmatics were outpatients and diagnosed by respiratory specialists from two hospitals in Wuhan between October 2010 and January 2012. The diagnosis of asthma was based on the presence of asthma symptoms and spirometry demonstrating airway hyperresponsiveness (Provoking Dose 20 (PD20) to methacholine <2.5 mg) and/or bronchodilator responsiveness (an increase of FEV_1_ more than 12% and 200 ml after inhaling 200 μg salbutamol) according to the Global Initiative for Asthma (GINA) guidelines^2^. We excluded the asthma patients who had any infectious diseases, heart diseases and other kinds of diseases such as hypertension, diabetes and cerebral vessel disease. A total of 1009 eligible asthmatics were collected in this study.

According to living area of the cases, the health controls without respiratory diseases, allergic diseases, infectious diseases, heart diseases and other kinds of diseases such as hypertension, diabetes and cerebral vessel disease were recruited from the same residential areas. A total of 1009 health controls were matched with asthmatics for gender and age (±5 years).

After excluded 227 participants for inadequate plasma samples, 910 asthmatics and 881 healthy controls were enrolled in the final analysis. All the asthmatics were divided into two categories by clinical manifestations of asthma, including 537 intermittent asthmatics and 373 persistent asthmatics. The intermittent asthmatics were defined as patients with asthma symptoms less than once a week, night waking due to asthma no more than twice a month and normal FEV_l_. Patients were identified as persistent asthmatics when they presented these clinical features exceeding the criteria of intermittent asthma. And the persistent asthmatics were classified into three categories such as mild (n = 246), moderate (n = 96) and severe (n = 31) asthma patients based on the clinical manifestations and the current level of treatment required to control symptoms and exacerbations, according to GINA guidelines^2^.

### Data collection

Basic data were collected by trained investigators via standard questionnaires by face-to-face interview, which covered information on socio-demographic and lifestyle such as education, occupational hazards exposure, family history of asthma, smoking, passive smoking and drinking status, physical activity, self-reported sleeping quality, and habits of keeping pets and planting flowers. Education level was categorized as low (below senior high school, ≤9 years), middle (senior high school, 9~12 years) and high (college degree or beyond, >12 years). Occupational hazards exposure was defined as whether individuals were exposed to industrial dust or chemicals in their workplaces. Physical activity was defined as regular exercise for more than 30 minutes at each time, at least once a week during the previous 6 months.

Clinical symptoms and signs of asthma patients such as cough, sputum, dyspnea, cyanosis and wheezing sound were collected by respiratory physicians. The cough status was classified into two types: occasional and chronic. The sputum status was classified into three categories: occasional, chronic and purulent. The occasional status indicated that the symptoms happen no more than three times once a week. The chronic status denoted that the symptoms happen last for more than two months. The degree of dyspnea was rated into 5-point (0 to 4) Grades according to modified Medical Research Council (mMRC) scale^32^. Then the severity of dyspnea was categorized as three types: mild (Grade 0 and 1), moderate (Grade 2) and severe (Grade 3 and 4).

Standing height and body weight were measured when individuals were standing with light indoor clothing and without shoes. Body mass index (BMI) was calculated as weight in kilograms divided by the square of height in meters.

### Ethics Statement

The study was approved by the Ethnics and Human Subject Committees of Tongji Medical School at the Huazhong University of Science and Technology (2011–17). The methods were carried out in accordance with relevant guidelines and regulations. All participants enrolled in this study were given written informed consents for participation, storage and use of blood samples.

### Spirometric examinations

The spirometric examinations were conducted when participants were in sitting position with a nose clip after normal breathing at least 5 minutes by an electronic spirometer (Chestgraph HI-101, CHEST Ltd., Japan). Each participant was required to accomplish three acceptable curves and the optimal data were selected for analyses according to the recommendations of the American Thoracic Society^33^.

### Inflammatory cytokines selection and determination

Approximately 5 mL fasting venous blood were collected in a tube with Ethylenediaminetetraacetic acid for each participant. On the same day of taking blood samples, the samples were centrifuged at 1500 rpm for 10 minutes at room temperature, then the plasma samples were isolated, aliquoted and stored at −80 °C until analysis.

In the screening stage, we randomly chose 8 asthmatics and 16 age- and sex-matched health controls from the participants without smoking or passive smoking status. The quantitative expressions of 280 cytokines (inflammation, growth factor, chemokine and other kinds of cytokines) in the plasma samples were measured by protein chip technology of Human Cytokine Antibody Array Q6000 (Raybiotech Inc., Georgia, USA, www.raybiotech.com). The cytokines were selected if they met one of the following criteria: 1) the plasma levels of cytokines were at least a 2-fold higher (fold change, FC ≥ 2) or less than half (FC ≤ 0.5) in asthmatic patients when compared with those in health controls, with the false discovery rate (FDR, q-value ≤ 5%); 2) the plasma levels of cytokines were significant differences (*P* < 0.05, 2-sided) between asthmatics and health controls analyzed by Student’s t-test. The total of 24 cytokines (HCC-1, IL-9, ICAM-2, IL-17, Adipsin, CA125, CA15-3, ErbB2, GROα, Nidogen-1, NSE, Thyroglobulin, NT-4, CCL11, I-309, Trappin-2, VCAM-1, DAN, DcR3, IFNα/β R2, TRAIL R1, sCD14, DR6 and XEDAR) were selected. According to an extensive literature review, the three inflammatory cytokines (IL-9, adipsin, CCL11), involved in Th17-related signaling pathway and considered as the pathogenesis of Th2-low phenotypes asthma, were selected to determine in all participants. Then, plasma levels of IL-17A, IL-9, adipsin and CCL11 in all participants were measured by enzyme-linked immunosorbent assay (ELISA) kits (IL-17A, adipsin and CCL11, R&D system; IL-9, eBioscience) according to manufacturer’s instructions.

### Statistical analysis

Socio-demographic and lifestyle characteristics were compared by Chi-square test for categorical variables and Student’s t-test for continuous variables. In the screening stage, differences of 280 cytokines antibody array data were analyzed using Significance Analysis of Microarray (SAM) 3.00 algorithm (http://statweb.stanford.edu/~tibs/SAM/index.html)^34^. SAM assigns a *d*-score to each cytokine on the basis of a two class paired analysis of expression changes and indicates significance by FC and q-value. The levels of four cytokines (IL-17A, IL-9, adipsin and CCL11) were not presented on normal distribution, so the differences in health controls, intermittent and persistent asthmatics were analyzed by kruskal-wallis-nemenyi test^35^. The correlations among these cytokines in asthma patients were analyzed by Spearman’s rank correlation. The associations between these four cytokines and adult asthma risk were evaluated by multivariate logistic regression models, with adjustment for potential confounders including age, sex, BMI, education, smoking status, passive smoking status, alcohol consumption, physical activity, family history of asthma, keeping pets, planting flowers, sleeping quality. In addition, we also divided the levels of four cytokines into two categorical variables such as normal and abnormal levels. The abnormal levels of IL-17A and IL-9 were more than the P_90_ levels of health controls, whereas the abnormal levels of adipsin and CCL11 were less than the P_10_ levels of health controls. After adjustment for age, sex, BMI and family history of asthma, the abnormal levels of IL-17A, IL-9, adipsin and CCL11 with adult asthma risk were analyzed by multivariate logistic analysis. The adjusted odds ratios (ORs) and 95% confidence (CIs) for the risk of adult asthma were calculated. The statistical analyses were computed by using SAS, version 9.3, software (SAS Institute Inc., Cary, North Carolina). Two-sided values of *P-*value < 0.05 were considered as statistically significance.

## Electronic supplementary material

Supplementary tables
