# Supplementary material for: Associations between Th17-related inflammatory cytokines and asthma in adults: A Case-Control Study
Source: Sci Rep. 2017 Nov 14;7:15502. doi: 10.1038/s41598-017-15570-8 (PMC5686108; doi:10.1038/s41598-017-15570-8)
Supplement: Supplementary file 1 — Supplementary tables [file 41598_2017_15570_MOESM1_ESM.doc]

**Associations between Th17-related inflammatory cytokines and asthma in adults: A Case-Control Study**

Ting Zhou^1,2#^, Xiji Huang^3,4,5#^, Yun Zhou^4,5^, Jixuan Ma^4,5^, Min Zhou^4,5^, Yuewei Liu^3^, Lili Xiao^4,5^, Jing Yuan^4,5^, Jungang Xie^6*^, Weihong Chen^4,5*^

**Affiliations:**

^1^Department of Occupational and Environmental Health, School of Public Health, Medical College, Wuhan University of Science and Technology, Wuhan, Hubei 430065, China

^2^Hubei Province Key Laboratory of Occupational Hazard Identification and Control, Wuhan University of Science and Technology, Wuhan, Hubei 430065, China

^3^Hubei Center for Disease Control and Prevention, Wuhan, Hubei 430079, China

^4^Department of Occupational & Environmental Health, School of Public Health, Tongji Medical College, Huazhong University of Science and Technology, Wuhan, Hubei 430030, China

^5^Key Laboratory of Environment and Health, Ministry of Education & Ministry of Environmental Protection, and State Key Laboratory of Environmental Health (Incubating), School of Public Health, Tongji Medical College, Huazhong University of Science and Technology, Wuhan, Hubei 430030, China

^6^Department of Respiratory and Critical Care Medicine, Tongji Hospital, Tongji Medical College, Huazhong University of Science and Technology, Wuhan, Hubei 430030, China

**^*^Corresponding authors:**

Dr. Jungang Xie

Department of Respiratory and Critical Care Medicine,

Tongji Hospital, Tongji Medical College,

Huazhong University of Science and Technology,

1095 Jiefang Avenue, Wuhan, Hubei, 430030, China

E-mail:xiejjgg@hotmail.com

Dr. Weihong Chen

Department of Occupational and Environmental Health,

School of Public Health, Tongji Medical College,

Huazhong University of Science and Technology,

13 Hangkong Road, Wuhan, Hubei, 430030, China

E-mail: [wchen@mails.tjmu.edu.cn](mailto:wchen@mails.tjmu.edu.cn)

**^#^** These authors contributed equally to this work.

**Supplementary Tables**

**Supplementary Table S1 Correlation coefficients of four cytokines in adult patients with intermittent asthma** (* *P* <0.01)

| Cytokines | IL-17A | IL-9 | Adipsin | CCL11 |
| --- | --- | --- | --- | --- |
| IL-17A | 1.00 |  |  |  |
| IL-9 | 0.04 | 1.00 |  |  |
| Adipsin | -0.15* | -0.19* | 1.00 |  |
| CCL11 | -0.04 | -0.12* | 0.34* | 1.00 |

**Supplementary Table S2 Correlation coefficients of four cytokines in adult patients with persistent asthma** (* *P* <0.01)

| Cytokines | IL-17A | IL-9 | Adipsin | CCL11 |
| --- | --- | --- | --- | --- |
| IL-17A | 1.00 |  |  |  |
| IL-9 | 0.03 | 1.00 |  |  |
| Adipsin | -0.19* | -0.21* | 1.00 |  |
| CCL11 | -0.09 | -0.12* | 0.38* | 1.00 |

**Supplementary Table S3 Adjusted odds ratios of persistent asthma by quartiles of cytokines in adults**

| Cytokines | Persistent asthmatics | Controls | Adjusted OR (95% CI) | *P*_trend_ |
| --- | --- | --- | --- | --- |
| IL-17A (pg/ml) |  |  |  | <0.0001 |
| Q1 (<1.70) | 47 | 267 | 1.00 |  |
| Q2 (1.70~3.64) | 98 | 214 | 7.68 (3.88, 15.22) |  |
| Q3 3.64~7.09) | 110 | 205 | 8.77 (4.36, 17.64) |  |
| Q4 (>7.09) | 118 | 195 | 5.79 (2.97, 11.29) |  |
| IL-9 (pg/ml) |  |  |  | <0.0001 |
| Q1 (<9.25) | 65 | 248 | 1.00 |  |
| Q2 (9.25~27.46) | 49 | 264 | 0.73 (0.45, 1.19) |  |
| Q3 (27.46~48.98) | 129 | 185 | 2.81 (1.83, 4.31) |  |
| Q4 (>48.98) | 130 | 184 | 2.56 (1.67, 3.93) |  |
| 1/Adipsin*10^-5^ |  |  |  | <0.0001 |
| Q1 (<16.99) | 34 | 280 | 1.00 |  |
| Q2 (16.99~25.08) | 50 | 263 | 1.55 (0.90, 2.67) |  |
| Q3 (25.08~40.05) | 84 | 230 | 3.18 (1.91, 5.31) |  |
| Q4 (>40.05) | 205 | 108 | 14.61 (8.79, 24.29) |  |
| 1/CCL11*10^-3^ |  |  |  | <0.0001 |
| Q1 (<14.27) | 57 | 257 | 1.00 |  |
| Q2 (14.27~21.91) | 59 | 254 | 0.95 (0.59, 1.54) |  |
| Q3 (21.91~33.18) | 85 | 229 | 1.40 (0.88, 2.22) |  |
| Q4 (>33.18) | 172 | 141 | 4.67 (2.96, 7.34) |  |

Adjusted for age, sex, BMI, education, smoking status, passive smoking status, drinking status, physical activity, family history of asthma, keeping pets, planting flowers, sleeping quality.
